# Wildlife parasitology: sample collection and processing, diagnostic constraints, and methodological challenges in terrestrial carnivores

**DOI:** 10.1186/s13071-024-06226-4

**Published:** 2024-03-13

**Authors:** Alicia Rojas, Nina Germitsch, Stephanie Oren, Alireza Sazmand, Georgiana Deak

**Affiliations:** 1 Laboratory of Helminthology, Faculty of Microbiology, University of Costa Rica, San José, 11501-2060 Costa Rica; 2 Centro de Investigación en Enfermedades Tropicales, University of Costa Rica, San José, 11501-2060 Costa Rica; 3 Pathology and Microbiology, Atlantic Veterinary College, University of Prince Edward Island, 550 University Ave, Charlottetown, PEI C1A 4P3 Canada; 4 Department of Anatomic Pathology, Faculty of Veterinary Medicine, University of Agricultural Sciences and Veterinary Medicine of Cluj-Napoca, 400372 Cluj-Napoca, Romania; 5 Department of Pathobiology, Faculty of Veterinary Medicine, Bu-Ali Sina University, Hamedan, 6517658978 Iran; 6 Department of Parasitology and Parasitic Diseases, Faculty of Veterinary Medicine, University of Agricultural Sciences and Veterinary Medicine of Cluj-Napoca, 400372 Cluj-Napoca, Romania

**Keywords:** Parasites, Wildlife, Post-mortem, Serology, Molecular detection, Scat, Carcass

## Abstract

Wild terrestrial carnivores play a crucial role as reservoir, maintenance, and spillover hosts for a wide parasite variety. They may harbor, shed, and transmit zoonotic parasites and parasites of veterinary importance for domestic hosts. Although wild carnivores are globally distributed and comprise many different species, some living in close proximity to human settlements, only a few studies have investigated parasites of wild terrestrial carnivores using non-specific techniques. Access to samples of wild carnivores may be challenging as some species are protected, and others are secretive, possibly explaining the data paucity. Considering the importance of wild carnivores’ health and ecological role, combined with the lack of specific diagnostic methodologies, this review aims to offer an overview of the diagnostic methods for parasite investigation in wild terrestrial carnivores, providing the precise techniques for collection and analysis of fecal, blood, and tissue samples, the environmental impact on said samples, and the limitations researchers currently face in analyzing samples of wild terrestrial carnivores. In addition, this paper offers some crucial information on how different environmental factors affect parasite detection postmortem and how insects can be used to estimate the time of death with a specific highlight on insect larvae. The paper contains a literature review of available procedures and emphasizes the need for diagnostic method standardization in wild terrestrial carnivores.

**Graphical Abstract:**

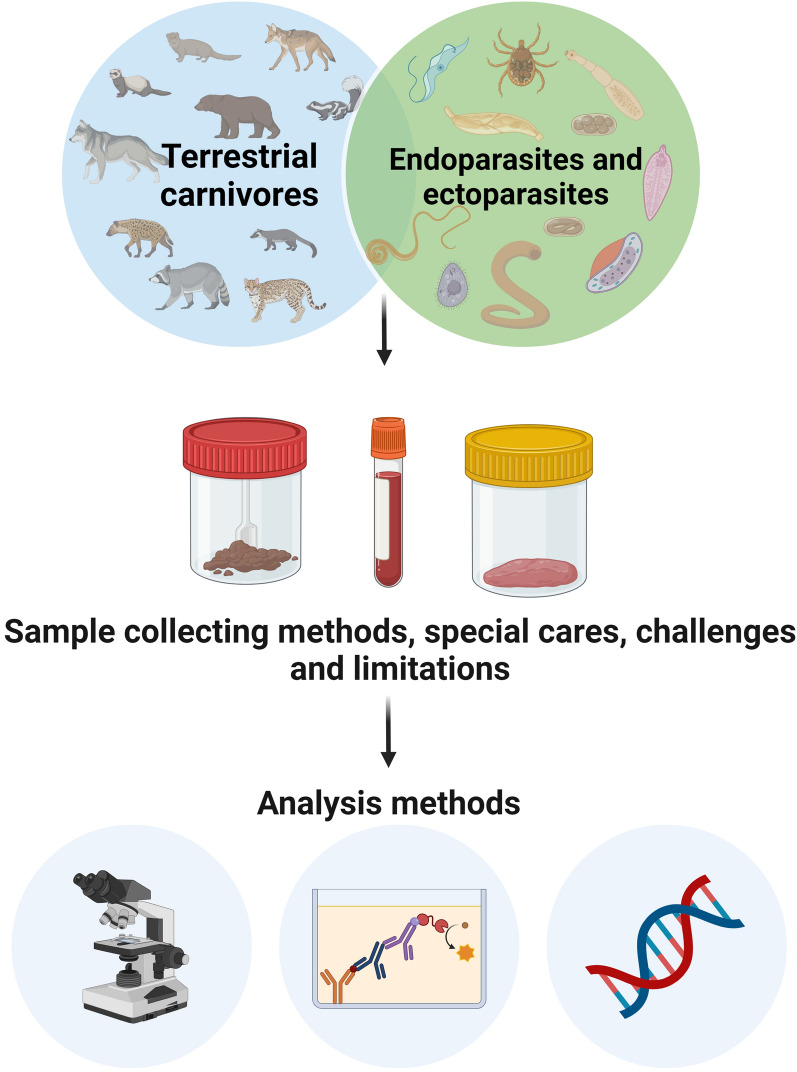

## Background

**Fig. 1:**
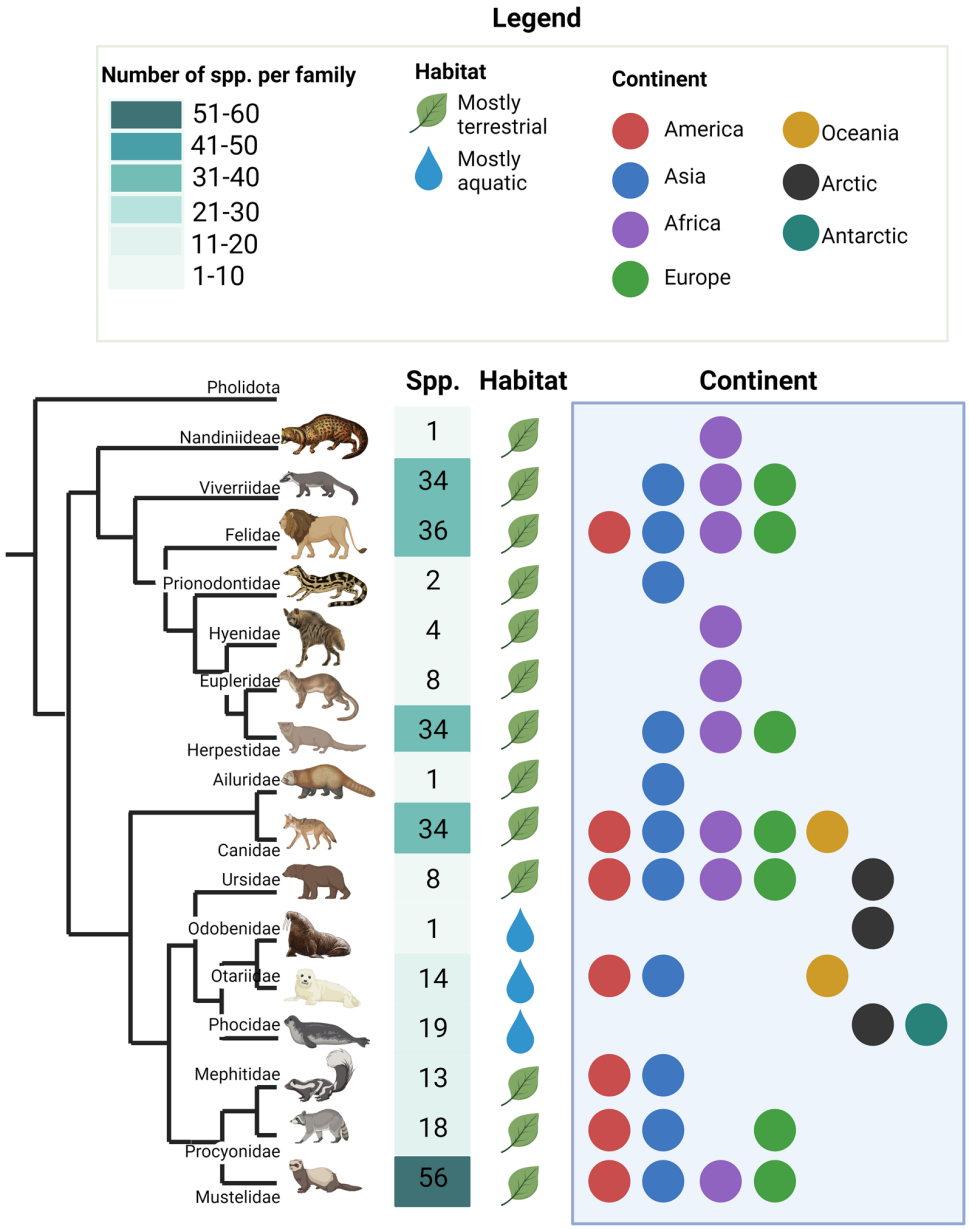
Carnivore families and their distribution. Phylogenetic representation of carnivore families based on previous analysis (Agnarsson et al. 2010; Hassanin et al. 2021), the approximate number of species per family, their terrestrial or aquatic habitat, and continent in which they are distributed. This figure was created using Biorender.com

Wild animals play a crucial role as reservoir, maintenance, and spillover hosts for a wide parasite variety (Fig. 1). They may harbor, shed, and transmit zoonotic parasites and parasites that are of veterinary importance for domestic hosts [1–3]. Environmental changes have led to the territorial expansion of some wild animal species, and their urbanization is increasing globally [4–6]. Other species are less adaptable to these changes, and thus, their numbers have declined [7, 8]. Consequently, a change in contact between wildlife, humans, and domestic animals has been observed in the last two decades, with increased contact in certain areas [9, 10].

Bridging infections between wild and domestic animals are becoming more frequent. Together with the movement and migration of wild animals, they have contributed and facilitated the spread of some parasites to new geographical areas [11–13]. The situation becomes more dire through the illegal trade of wild animals and the introduction of invasive wild mammals to regions where they contribute to zoonotic parasite transmission [14–16]. Such spillover events were observed for the lungworms *Angiostrongylus vasorum,* whose geographical expansion directly correlates to its main canid host, the red fox (*Vulpes vulpes*) [12] and *Crenosoma vulpis,*, which was recently reported in northern Africa and associated with the expansion of red foxes in the African continent [17]. In addition, some parasites of veterinary importance have been reported in wild animals in new geographical areas, mainly in carnivore hosts, presenting a risk of spread or spillover to domestic species [18–21]. Likewise, some non-native animal species, such as raccoons (*Procyon lotor*) in Europe, are responsible for the introduction of foreign parasites, such as the American ascarid *Baylisascaris procyonis*, now a new zoonotic nematode in Europe [22]. Although wild carnivores are globally distributed and comprise a high number of different groups living close to human settlements (Fig. 1), only a few studies have investigated parasites of wild terrestrial carnivores using comprehensive procedures [23, 24]. Access to samples of wild carnivores may be challenging as some species are protected and endangered, and others are elusive and prefer to stay hidden, possibly explaining the data paucity. So far, methods such as macroscopic examination, serological tests, and molecular tools have been employed for parasite detection in organs, tissues, and body fluids from wild hosts [25].

Considering the significance of wild terrestrial carnivore species as spreaders and reservoir hosts for parasites of veterinary and public health importance, combined with the lack of precise diagnostic methods, this review aims to offer an overview of the diagnostic approaches for parasite investigation in these hosts. The paper reviews specific techniques for the collection, preservation, and analysis of fecal, blood, and tissue samples, the environmental impact on said samples, and the limitations researchers currently face in analyzing samples of wild terrestrial carnivores.

## Analysis of fecal samples

Fecal specimens may render significant information about parasites’ identity, diversity, ecology, and epidemiology, as well as infection intensity, dynamics, and distribution. In addition, it may help to understand possible threats to carnivore populations, evaluate the impact of parasitism on population dynamics, the animals’ dietary habits, host population estimates, breeding, resource selection, and partitioning, and manage effective control and conservation strategies [26]. However, capturing and handling wild animals can often be challenging due to their wariness toward humans, trained personnel, and the costs [27]. Therefore, non-invasive sampling is often used, and molecular scatology has become a frequently used technique to study all the above features [26].

A multi-evidence approach should be conducted to monitor the presence of an animal species and to avoid identification and multiple sampling biases [28]. Identification bias refers to the possible misidentification of a species based on the morphological assessment of a scat, whereas repeated sampling bias refers to the possibility of sampling a scat from the same individual more than once [29]. This first step of animal species identification has crucial epidemiological and ecological implications. This is the case when describing a new animal species as a host for a certain parasite [10]. Herein, we provide several aspects to consider when collecting and analyzing scat or fecal samples.

### Fecal sample collection, preservation, and identification

Different sample collection methods have been described, each one having its advantages and disadvantages in terms of costs, risk of pathogen transmission to the researcher, and avoiding repeated sampling bias, i.e., sampling the same individual twice or more [29]. Invasive monitoring involves the trapping of animals and the collection of samples directly from the animal’s rectum by using a disposable glove or a fecal loop [29, 30]. Alternatively, invasive sampling may also be performed by taking feces directly from the gut of carcasses [31]. Furthermore, the necessity of capturing animals for sample collection should be argued and protocols should be prepared and presented in advance to receive permission from an ethical committee. For these reasons, non-invasive approaches have increasingly been used [29, 32, 33].

During invasive monitoring, repeated sampling bias is avoided, but this strategy may involve animal euthanasia or increase animal stress when sampling from the rectum and is costly. Therefore, invasive sampling is often used for large spatial analysis or long-term monitoring or when animals are marked with GPS collars [34]. Moreover, fecal samples collected directly from animal carcasses require special care to avoid transmission of zoonotic pathogens [35]. For instance, work surfaces should be prepared and sterilized accordingly, adequate personal safety equipment should always be used and carcasses must be frozen at −80 °C for at least 3 days [36]. Even though freezing decreases the detection of some parasites due to their sensitivity to low temperatures, this procedure ensures a reduction in the risk of pathogen transmission. After the elapsed time, ectoparasites, as well as tissue or fecal samples, may be collected. If the description of gastrointestinal parasite communities is aimed, the entire small intestine and ceca should be examined by segmenting the whole gut into equal parts with further microscopic analysis [36]. This task may be lengthy and laborious, but some methods have eased the collection of macroscopic parasites. One protocol known as “shaking in a vessel technique” involves the longitudinal opening of the gut with the release of all its contents into a plastic container equipped with a 100–200 µm diameter sieve in the cap. Then, gut contents are washed with abundant water, and parasites are sieved and collected [37].

**Fig. 2:**
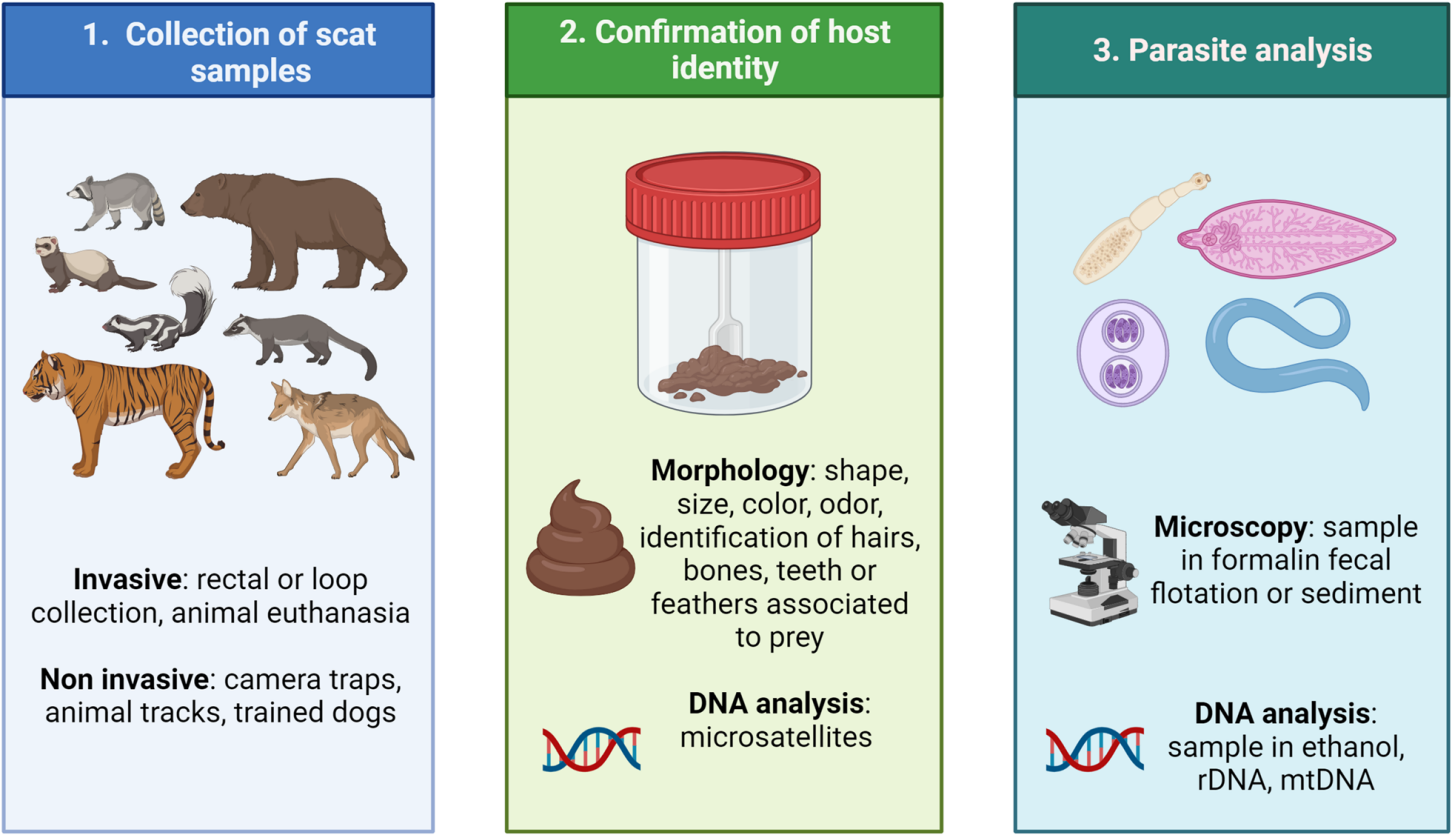
Basic procedures for the analysis of parasites in fecal samples from wild terrestrial carnivores. This figure was created using Biorender.com

Non-invasive sampling involves collecting scats from the environment and detecting animals with camera traps, analysis of footprints (Fig. 2a) [27, 30], or the use of trained scat-detection dogs as previously used for coyote (*Canis latrans*), jaguars (*Panthera onca*), cougars (*Puma concolor*), ocelots (*Leopardus pardalis*), or cheetah (*Acinonyx jubatus*) scats [29, 38–41]. The latter approach may be faster and more specific but more costly than the two former methods. Previous studies have compared different non-invasive sampling strategies regarding the latency of initial detection of the carnivore and the probability of detecting the animal. All methods showed varying efficiencies for different carnivore species [27]; therefore, it is recommended to consider the target animal host when selecting a sampling method.

**Fig. 3:**
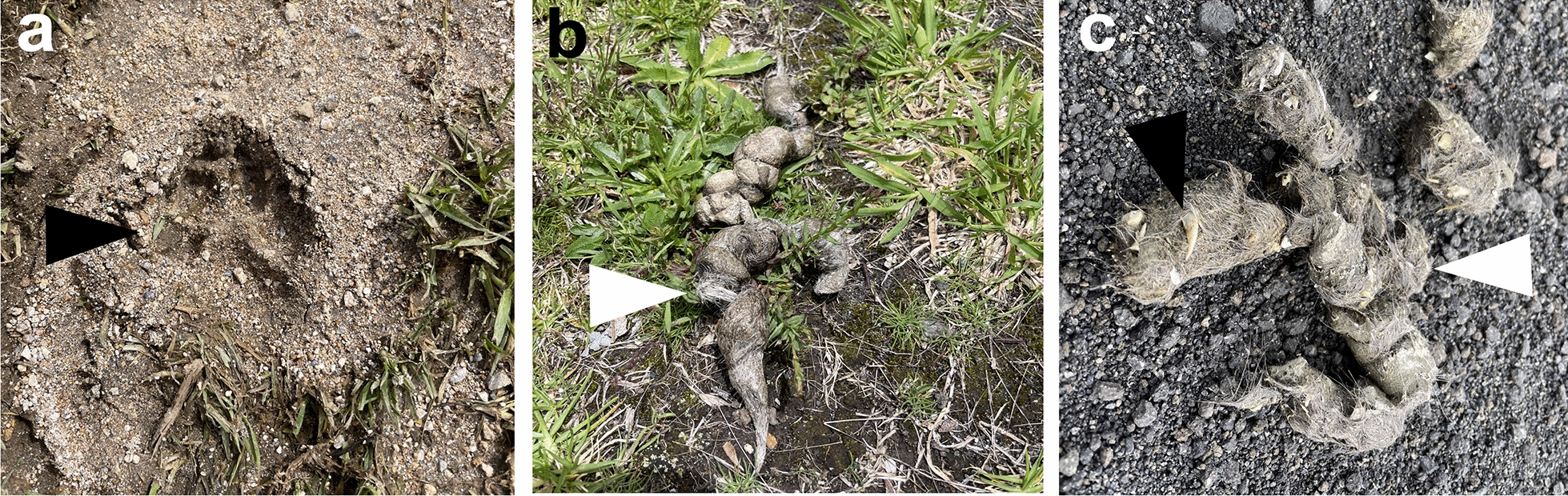
Examples of wildlife sampling. **a** Foot pad of a coyote (*Canis latrans*). **b** Hair remnants (white triangle) associated with prey in a scat from a coyote. **c** Hair (white triangle) and nail (black triangle) remnants associated with prey is a scat from a coyote

The aim of the study should be defined before collecting and preserving scats since some preserving agents may prevent further analyses and may alter the analysis of certain specific parasitic stages (Fig. 3). Fresh fecal samples are preferred since species assignment by using morphological or molecular methods is more accurate [26]. Room temperature storage will be convenient if samples are analyzed within 24 h since deoxyribonucleic acid (DNA) degradation will start and parasite viability will decrease [42, 43]. For instance, the first (L1), second (L2), and third (L3) larval stages of some nematode species of the families Ancylostomatidae and Strongyloididae have positive geo- and thermotropism which enables them to be concentrated in a Baermann apparatus [44]. Therefore, false negative results would arise if samples aimed at the detection of parasites of these two families were immediately frozen or dried. Furthermore, fecal samples kept at room temperature for more than 24 h with low humidity will be useful only for helminth egg or oocyst analysis, similar to the conditions found in the study of coprolites [45]. On the other hand, analysis of fecal samples collected after > 3 days in a high-humidity environment may contain degraded larval forms [46], as observed for strongylids of the white-tailed deer (*Odocoileus virginianus*), and can also stand true for carnivore-associated parasitic larvae.

Samples maintained and processed at room temperature for periods less than 24 h may still be suitable for morphometric measurements of tapeworm segments and adult nematodes when/if present. It is recommended to place fresh worms collected from feces or any animal cavity in warm phosphate-buffered saline (PBS) buffer or saline solution so their tissues relax while they lose their viability [47]. Alternatively, other protocols recommend placing fresh worms in a container with tap water and then refrigerating and storing them in ethanol or formalin, depending on the aims of the study [48]. On the contrary, if worms are placed directly in ethanol or cold PBS their muscle fibers will contract, and some major taxonomical structures will not be evident, or their measurements will vary from type specimens (personal observations of the authors). For detailed protocols regarding nematode, trematode, or cestode clearing and staining, consult Sepulveda and Kinsella [47].

DNA degradation increases with time and higher temperatures, therefore, samples aimed for molecular analysis should be collected and stored at −20 °C whenever possible. Still, frozen feces may be thawed and analyzed for the presence of helminth eggs or some larvae families. It has been observed that *Crenosoma vulpis* larvae retain their viability after 7 months at −20 °C and at −80 °C they survive for at least 50 days] [49, 50]. Larvae of *Crenosoma goblei* have been reported to survive freezing at −25 °C for up to 14 months [51]. It is important to keep in mind that although freezing is suitable in most cases, this process may destroy the fine structures of host tissues and parasites. Generally, histopathological examination, helminth staining, and examination using electron microscopy are almost impossible from frozen tissues [52]. Hence, if macroscopic parasites are observed in scats, some may be separated from the sample and relaxed in warm PBS for morphological assessment and some others in 70–95% ethanol for molecular analysis or frozen at −20 or −80 °C optimally in DNase-free tubes. Scats may be placed in formalin (maximum 4%) for long-term preservation and fixation of parasites. Nevertheless, formalin fragments DNA and creates adducts that halter further molecular reactions [53]. For this reason, freezing in 70–95% ethanol [32] or storing in dimethyl sulphoxide saline solution at a proportion of 1:4 scat:solution [40, 54, 55] is recommended to prevent DNA degradation.

Correct identification of host animal species is crucial for parasite identification. Misidentification may be diminished with morphological observations of scats and DNA analysis of samples. Analysis of macroscopic characters includes color, size, shape, and presence of undigested animal or plant matter such as bones, feathers, teeth, hair, scales, exoskeletons, or otoliths (Fig. 2b and c) [56, 57]. Morphology-based identification is inexpensive and does not involve specialized equipment [58, 59] but requires high expertise in the identification of the aforementioned elements and the freshness of samples may significantly alter these characteristics. For instance, high humidity found in tropical locations may disintegrate some of these diet-associated elements, in contrast to low humidity [60]. In addition, the risk of repeated sampling is higher in morphology-based identifications but can be reduced with the rule of minimum distance using the estimated home range size of the animal [56, 61]. Therefore, molecular biology-based approaches are becoming widely used due to their high accuracy and decreasing costs [26].

Several factors may interfere with the correct species assignment during scat morphological analysis. Skilled scat surveyors from Scotland could not consistently identify scats of American minks (*Neogale vison*) based on footprints, fecal sample size, shape, and smell [62]. The identity of these samples was confirmed by extracting the DNA of scats, which were confirmed to belong to pine martens, red foxes, polecats, and stoats [62]. A study on scats from snow leopards (*Panthera uncia*) in Pakistan showed that only 52% of scats were correctly assigned when doing a morphological assessment, whereas the rest of them belonged to red foxes, gray wolves (*Canis lupus*), and corsac foxes (*Vulpes corsac*) [63]. In addition, carnivores with similar body sizes and diets may have overlapping scat measurements, and thus, can be misidentified. For instance, fecal samples of pine martens and red foxes from Britain could not be identified in the field with high confidence and were mistakenly classified when confirmed with DNA analyses [64]. However, scat identification may sometimes prove unequivocal when no other sympatric species are present, like in the case of brown bears (*Ursus arctos*) in European countries. Even though morphology-based scat identification methods seem convenient in instances of limited resources, these may lead to false identifications, especially if sympatric carnivore scats are screened, thus DNA analysis should be aimed for [26].

### DNA analysis of fecal samples

This should be performed whenever possible due to the high accuracy in host and parasitic species assignment, and their application in non-invasive fecal sampling [28]. Host DNA is detected from epithelial cells shed from the gastrointestinal tract of the predator. In this way, predator DNA exceeds prey and parasitic DNA. Still, high loads of bacterial DNA and polymerase chain reaction (PCR) inhibitors remain in the sample. Mitochondrial DNA (mtDNA) like cytochrome b (*cytB*) or *12S* [26, 36, 55] or single nucleotide polymorphisms (SNPs) [29, 65] are widely used for predator identification. Mitochondrial DNA is present in hundreds to thousands of copies per cell, whereas there are only two nuclear DNAs per cell [66]. Moreover, SNPs may render useful information regarding population structure, hybridization, or individual relatedness [36, 65]. Nevertheless, DNA analysis may be challenging due to the non-controlled conditions of collected samples.

Repeated sampling bias is decreased in DNA analysis by genotyping individuals with microsatellites [32] or SNPs [29, 30, 65]. SNP analysis amplifies short DNA fragments, which enables higher amplification rates when compared to using longer and fragmented DNA, whereas microsatellite amplification has low success and high error rates due to the low quantity and quality of target DNA [65].

Special care should be taken when handling samples for DNA analysis. Quantity and quality of DNA may be affected by the time scats remain in the field, direct exposure to sunlight, high humidity and temperatures, increased hemoglobin content derived from the animal’s diet, drying or preservation method after collection, and DNA extraction method [67]. All these factors may increase the activity of exo- or endonucleases of bacterial origin that degrade DNA or increase the activity of PCR inhibitors present in fecal samples, leading to low DNA concentration [65]. Therefore, drying scats are usually performed to decrease the activity of nucleases and DNA degradation. Detection of nematode non-embryonated eggs in feces may contain lower DNA and when feces are fresh, in vitro embryonation is recommended as the presence of larva in the eggs was associated with higher DNA quantity, so this could be done for reducing the false negative results [68].

Some research teams opt to dry scats when collected to reduce moisture from feces and, thus, bacteria-induced DNA degradation. However, this step is optional since successful DNA amplification has been achieved from non-dried samples [65]. Drying can be accomplished with silica desiccants, or by air [32, 63], freezing, heating at 40 °C in an oven [29, 30], or microwaving. Freezing and oven-drying have been the most effective methods for subsequent host DNA or parasitic DNA amplification [66]. Once samples are dried, the outer layer of the scats is scraped for further processing [29]. DNA extraction may be done with commercial kits specific for feces [26, 40, 55, 65, 67].

### Parasite analysis of fecal samples

This may be accomplished with two different strategies depending on the employed sample preservation agents. Coproparasitological analysis including fecal flotation, ethyl-formalin concentration, sedimentation, and larval cultures may be done on unpreserved samples or those preserved in formalin or ethanol, whereas molecular studies can be done only on samples stored in ethanol or no preservative [69].

Fecal flotation, sedimentation, and larval concentration methods have been used to detect parasite eggs, larvae, and oocysts. Protozoan cysts or trophozoites are difficult to observe in scats since their morphology is significantly altered during the time elapsed from collection to analysis. For instance, flotation using saturated sodium chloride solution was used to detect eggs and oocysts from Formosan black bears (*Ursus thiberanus formosanus*) of Japan, leading to 77.3% positivity with the following parasites: *Baylisascaris transfuga*, *Strongyloides* sp., *Trichostrongylus* sp., *Oesophagostomum* sp., ancylostomatids, *Taenia* sp., *Physaloptera* sp., *Gongylonema* sp., and *Cryptosporidium* sp. [56]. In addition, *Capillaria* spp., ancylostomatids, *C. vulpis*, *A. vasorum*, *Toxocara canis*, *Sarcocystis* spp., *Hammondia*/*Neospora* spp., *Cystoisospora ohioensis*, *Giardia* sp.,*Cystoisospora canis*, *Trichuris vulpis*, *Taenia* spp., *Dibothriocephalus latus*, *Strongyloides* spp., *Opisthorchis felineus*, *Toxascaris leonina*, *Alaria alata*, and *Mesocestoides litteratus* were detected in Croatian wolf scats using concentration with sodium acetate-acetic acid-formalin (SAF) and ethyl acetate [70]. Moreover, a combination of zinc salt sedimentation–flotation was used to detect ascarids, strongylids, taeniids, *Trichuris* sp., *Capillaria* sp., *Eimeria* spp., and spirurids from red pandas of Nepal [61]. A previous work described both flotation and sedimentation to increase the detection of parasites in wildcats (*Felis silvestris*) from Italy showing that 90.9% of samples were positive by the flotation technique, 65.4% positive with sedimentation and 60.9% of samples were positive when using both assay [33].

Considering that carnivores eat other animal tissues as prey, spurious or false parasitism should be distinguished from true parasitism [44]. Usually, spurious parasitism is suspected when observing egg or oocyst species unusual in the studied carnivore and results from the ingestion of infected tissues or feces and the passing of their eggs or oocysts through the gastrointestinal tract [71]. Examples are the observation of rodent-associated *Hymenolepis diminuta* eggs in coyotes from Costa Rica [72], *Habronema* sp., *Schistosoma* sp., *Eimeria felina*, and *Macracanthorhynchus hirudinaceus* eggs in African lions (*Panthera leo*) from Tanzania [71], *Dicrocoelium dendriticum* and *Trichuris* sp. eggs in brown bears from Spain [57], lagomorph strongylid *Protostrongylus pulmonaris* originating from preys were observed in Eurasian lynx (*Lynx lynx*) from Germany [73]. Larval concentration is less often employed but can be accomplished in unfrozen freshly collected samples since larvae remain viable and their morphology remains preserved only for a few hours [56]. A larval culture method, also known as the Harada-Mori technique, was used for the detection of larvae in Formosan black bears (*Ursus thibetanus formosanus*) [56] and the Baermann method has been used for the recovery of *A. vasorum* larvae on scats of red foxes from Canada [74]. Nevertheless, parasite analysis remains challenging since scats are usually dried and trophozoites or larvae have lost their motility.

Paleoparasitological techniques dealing with samples that have suffered extreme desiccation and fossilization are suitable for old, and dried fecal specimens. In archaeological materials, helminth eggs, larvae, and oocysts can be successfully recovered and identified with rehydration, homogenization, filtering, and sedimentation. This was the case of the recovery of Strongylida eggs and Eucoccidiorida oocysts from a coprolite of a carnivore mammal from Brazil [75]. In another study from Argentina, eight different nematode species were found in coprolites of carnivores, presumptively *Helminthoxys* sp., *Physaloptera* sp., *T. leonina*, *Trichuris* sp., *Heteroxynema viscaciae*, and other Spirurid and Oxyurid eggs [76]. This demonstrates the utility of simple and inexpensive coproparasitological methods for the recovery of parasitic stages from extremely dried fecal samples as old as 11,000 years before the present. Nevertheless, molecular parasitic identification remains challenging in these samples, since DNA amplification may not always succeed [75], although *T. leonina* *cox*1 fragment could be amplified from a cougar coprolite dated from the Pleistocene 16,573 to 17,002 years ago [77].

In the recent decade, detection of genomic material of parasites by end-point PCR, real-time PCR, or metabarcoding has become popular [78]. Molecular analysis is especially useful in the differential diagnosis of taxa whose diagnostic stages cannot be identified to species, such as the case of taeniid, strongylid, or ancylostomatid eggs. Therefore, multiplex PCRs with amplicon sequencing directed to the mitochondrial DNA of *Taenia* spp. and *Echinococcus* spp. have been designed [79] and employed in field studies. A study in wolves, red foxes, corsac foxes, and snow leopards from Mongolia revealed that 27.7% of scats were positive for *Taenia hydatigena* and *Mesocestoides* spp. by running conventional PCRs that amplified 400 base pairs (bp) cytochrome c oxidase I (*cox*1) and 314 bp *12S* fragments [80]. *Taenia hydatigena* and *T. multiceps* DNA were detected in Croatian wolf scats by using semi-nested cestode universal PCR [70]. Furthermore, *Echinococcus granulosus* sensu lato, *Echinococcus canadensis*, *Echinococcus multilocularis*, *Taenia serialis*, *Dipylidium caninum*, and other unidentified *Taenia* and *Mesocestoides* spp. were detected in wolf scats from the USA by using a multiplex PCR for the detection of cestode species [78].

### Coproantigen testing

Coproantigen tests allow the detection of parasite antigens in feces. Current laboratory and commercially available tests are largely genus specific and have high specificity. Advantages of coproantigen testing include antigen detection prior to prepatency and detection is independent of egg or oocyst output. Detectable parasite antigens usually remain stable for several days to months, depending on storage temperatures (−80 °C to 35 °C), allowing for coproantigen testing for an extended period of time [81]. In wild terrestrial carnivores, coproantigen tests have successfully been employed to detect *E. multilocularis* [82, 83], *E. granulosus* [84], *Giardia* sp., *Cryptosporidium* sp. [85], and hookworm [86]. However, cross-reaction between closely related taxa remains challenging [87].

## Analysis of blood samples

**Fig. 4:**
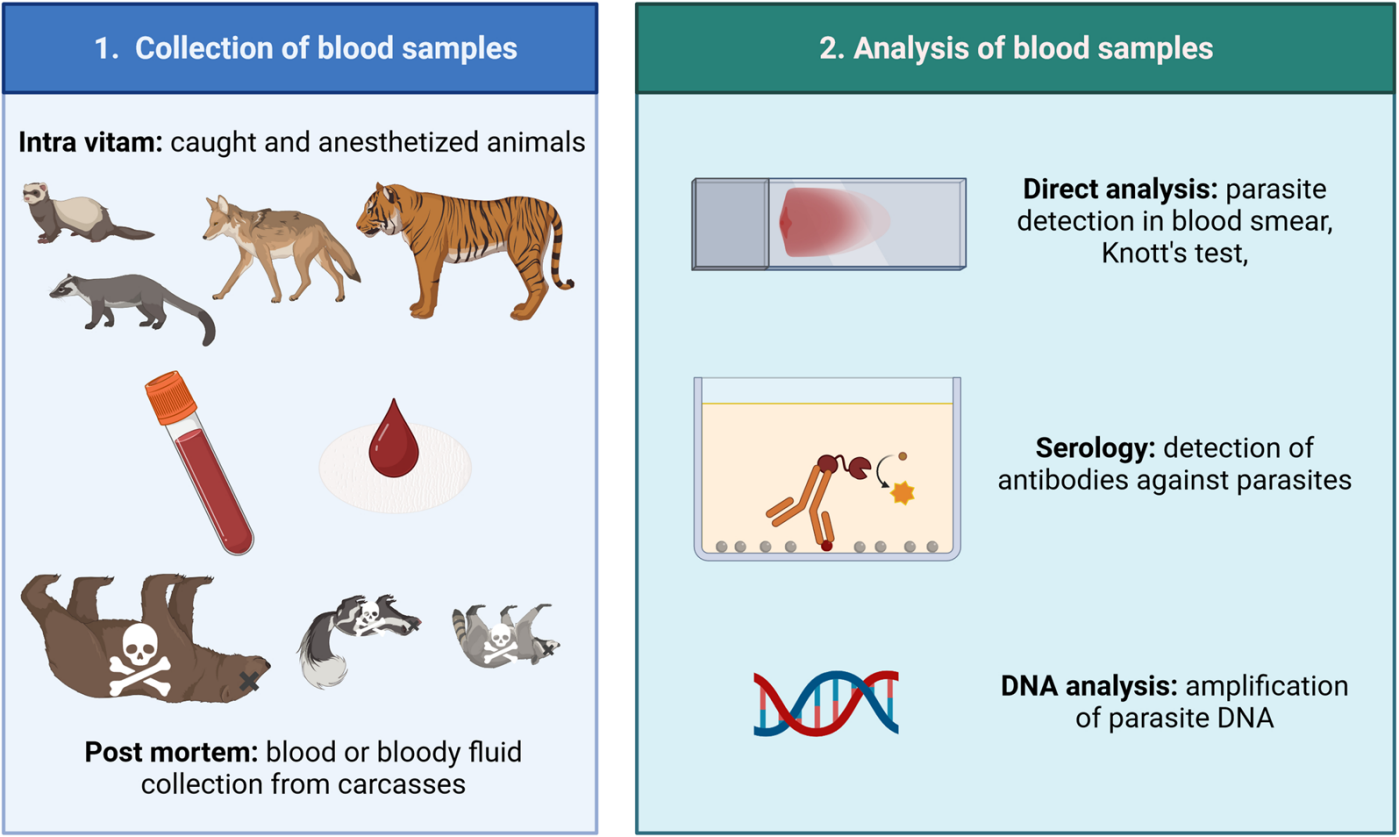
Basic procedures for the analysis of parasites associated to blood samples from wild terrestrial carnivores. This figure was created using Biorender.com

In contrast to scats, analysis of blood samples of terrestrial carnivores does not have complications related to host identification (Fig. 4). Moreover, the parasites’ diversity of the host animals or its higher taxa is largely known. Blood is the ideal tissue for examination of infection with vector-borne pathogens including protozoan parasites e.g., piroplasms (*Babesia* and *Cytauxzoon*), *Hepatozoon*, *Trypanosoma*, filarial nematodes with blood-dwelling microfilariae, e.g., *Dirofilaria*, *Acanthocheilonema*, and blood-borne bacteria, e.g., *Anaplasma*, *Ehrlichia*, *Rickettsia*, haemotropic *Mycoplasma* species, *Bartonella*, and more [88, 89]. Blood samples can furthermore be used for serological testing of a variety of different parasitic diseases.

### Blood sample collection and preservation

Sample collection from wild animals may not always be straightforward and there are currently no standardized sample collection protocols for blood samples. The collection of blood samples from live wild carnivore species is challenging but possible when the animal is trapped or caught in nature or when captive animals are under anesthesia or sedation for veterinary procedures. Blood samples are occasionally collected from wild carnivores housed in captivity for routine health checkups, e.g., for evidence of anemia, infection, or inflammation—via hematological parameters—and levels of electrolytes, as well as to assess organ function [90]. Samples collected for these purposes may also be used for parasite screening [91]. In the field, blood and serum samples can be collected from trapped wild animals. To collect adequate blood samples from terrestrial carnivores they must be anesthetized via remote injection or live captured during catch-and-release or relocation operations [92–94]. However, direct sample collection from wild animals can sometimes be considered unethical because human proximity to wildlife may cause flight response and lead to abandonment of offspring, interruption of food intake, or may even lead to injuries to the animal that is to be caught, other animals in proximity, and occasionally humans. Blood samples collected under these circumstances are usually interventions after observation or susceptibility to an outbreak or due to other interventions [95, 96].

Frequently, blood samples of wildlife are collected postmortem from carcasses in the field or in the laboratory from animals that either died, were hunted or trapped, or road killed. When death occurs, veins and arteries collapse and blood starts clotting and hemolyzing, which makes using syringes nearly impossible. However, occasionally non-coagulated blood can be collected from large veins such as the femoral vein and auxiliary vein. Blood can also be collected from other locations in carcasses in various states. Most of these samples are suitable samples for a variety of different tests. Whole blood samples can be collected from the heart or large blood vessels, they may be liquid or made up of partially or fully coagulated blood [97, 98]. Fluid collected from the thoracic cavity during necropsy is frequently collected and referred to among others as serosanguineous fluid, body fluid, thoracic transudate, bloody fluid, or bloody body fluid [99, 100].

Optimally, blood samples should be collected from fresh carcasses before postmortem changes as autolysis affects the sampling process and diagnosis. Whole blood can be collected in tubes directly and centrifuged to collect serum, it can be frozen for further later processing or collected into tubes containing anticoagulants such as ethylenediaminetetraacetic acid (EDTA) to attempt to separate plasma by centrifugation for further testing. However, the chance of successful plasma collection depends on the time since death, geographical region (e.g., Iceland versus Kenya), and the season (e.g., freezing winter versus hot-humid summer). Even if blood component separation is not possible, EDTA-mixed-blood can still be used for consequent DNA isolation when stored at −80 °C. Alternatively, blood can be stored in tubes with 70% ethanol or frozen. Preferably, even the EDTA or ethanol-preserved samples should be frozen for long term storage [101].

When samples have been collected in the field, particularly in remote areas, from either live animals or carcasses, sample integrity can be compromised due to fluctuating temperatures, extended improper storage, and uncontrolled and prolonged transport conditions. To overcome some of these potential complications technologically advanced cards and filter papers are being used for transport and storage of infectious samples for molecular diagnostics and serology. The cards are cotton-based, cellulose paper impregnated with a mix of chemicals that lyse cells, denature proteins, and protect DNA, leaving a sample suitable for molecular identification without the risk of disease contamination and suitable for long-term storage [102, 103]. Several other filter paper types have been used to store whole blood or serum samples for serology. Dried samples result in comparable sensitivity and specificity to liquid serum samples in most serological tests if stored properly and if elution of dried samples is comparable to serum [104]. Convincing results have been obtained with PCR, enzyme-linked immunosorbent assays (ELISA), and even card agglutination test for *Trypanosoma* (CATT) when blood, buffy coat, serum, or plasma were recovered from filter papers [105]. The use of filter paper is fairly simple, they are soaked in blood and air-dried. Best results are obtained if filter paper is stored in a sealed bag with a desiccant immediately after drying [104]. Filter paper blood collection can be applied by researchers, veterinarians, hunters, trappers, and trained laypeople [106, 107]. Filter paper samples do not require refrigeration after collection, they are lightweight, and they can be easily transported and mailed in regular envelopes using standard mail at room temperature [108]. If any means of preservation is accidentally not available, the small volumes of blood for molecular examination can be dried on normal filtration paper, soft tissue paper, toilet paper, or similar [52].

### Microscopic examination

When fresh blood specimens are available, different methods including wet blood smear, hematocrit concentrations technique (HCT) or buffy coat method (BCM), and mini anion-exchange centrifugation technique (mAECT) can be employed to observe different parasitic forms of blood parasites under the microscope. However, morphological characteristics of parasites within one genus are not always distinct and make differential diagnosis nearly impossible [105, 109, 110]. For instance, intraerythrocytic merozoites of three canine *Babesia* species, including *Babesia rossi* (Nuttall 1910), *Babesia canis* (Pianna and Galli-Vallerio 1895), and *Babesia vogeli* (Reichenow 1937), are morphologically identical when examined by light microscopy [111]. Moreover, the analytic sensitivity of microscopical examination methods is lower than molecular-based techniques, e.g., 1 × 10^4^–1 × 10^5^ parasites/mL for wet blood film versus 1–10 parasites/mL for genus/species-specific primers in the case of trypanosomes [105], and they work mainly (sometimes only) when used during the acute phase of infection [112]. It is worth mentioning that microscopic examination can have advantages over molecular analysis, e.g., in the case of coinfections, PCR using group/genus/species-specific primers, which are very common, and even Sanger sequencing can fail when the ratio of parasitemia is in favor of one parasite [113]. Moreover, cross-species reactivity is a known phenomenon in different parasite taxa, e.g., *Leishmania* [114], *Trypanosoma* [110, 115], and *Dirofilaria* [116].

### Molecular identification

With the advancement of molecular methods and their widespread use, especially in the 21st century, molecular-based techniques that have higher analytic sensitivity have become more popular. Hence, when molecular-based tools are available, PCR and its variants, e.g., multiplex PCR, nested PCR, and quantitative PCR should be preferred. However, the costs of molecular testing are higher compared with microscopy and it needs specialized laboratory equipment and skilled personnel [110]. In blood samples, similar to fecal samples (see “DNA analysis of fecal samples” section) autolysis or self-digestion degrades DNA and activates PCR inhibitors leading to lower DNA concentrations. DNA extraction methods may be chosen among established techniques considering the available equipments, budget, and time [117].

### Serology

Serological methods allow the detection of antibodies in serum and other body fluids. They can detect current infections or previous exposure to pathogens, infectious agents, or foreign proteins. Some methods have been developed to detect pathogen antigens [118]. Antibodies, however, usually remain in the body longer than antigens [119]. Serological methods have long been used to study the infection history of wild animals and for the detection of parasitic pathogens in wildlife, because parasite isolation from wildlife can be challenging even under ideal conditions [120]. Recovery of some parasites would require invasive methods or even lethal sampling, for such and many other parasitic infections collection of blood or other samples for the detection of antibodies or antigens is often favorable. Furthermore, antibodies can persist for a long time, occasionally longer than the parasitic agent [120–123]. Single samples can be tested, or tests can be implemented for screening of large wildlife populations [21, 120]. An advantage of serological testing for screening over parasite isolation is that large numbers of samples can be tested at once in a short period of time. It is advantageous for surveillance and epidemiological screening of populations.

An advantage of serological testing for parasite exposure postmortem is that the carcass state often does not hinder sample collection and diagnosis. Blood samples and serosanguineous fluid can still be collected from carcasses and organs of wildlife that were stored for extended periods of time, that were frozen, or exposed to heat or partial desiccation. Collected blood samples can be stored after collection for decades at −20 °C or below without significant disintegration of antibodies [124]. However, extended improper storage and repeated freeze–thaw cycles may impact the stability of antibodies and antigens in samples [125]. Sample collection method and sample type,, however may lead to differing serological results. Thoracic transudate outperformed blood clots in certain serological tests [98] and filter paper samples are diluted in an elution solution, due to this dilution factor, and low antibody levels likely are not detected [107].

#### Different serological tests used in wild terrestrial carnivores

A variety of different serological tests have been developed for the detection of exposure to all classes of parasites. Some of the most applied techniques for samples of wild terrestrial carnivores are ELISAs, agglutination tests, immunofluorescent antibody tests (IFAT), and immunoblotting [94, 97, 126].

A range of laboratory-developed and commercially available tests have been evaluated for use in wildlife. The serological detection of *Toxoplasma gondii* via an agglutination test has been implemented for many wild animal species. Aside from the agglutination test, ELISA and IFAT have also been used or evaluated for the detection of *T. gondii* antibodies in wild terrestrial carnivores; some of the tested animal species include arctic foxes (*Vulpes lagopus*) [126, 127], black bears, wolves [128], polar bears (*Ursus maritimus*) [94], Eurasian lynx [129], cheetah, African lion, leopard (*Panthera pardus*), caracal (*Caracal caracal*), brown hyena (*Hyaena brunnea*), spotted hyena (*Crocuta crocuta*), black-backed jackal (*Canis mesomelas*), honey badger (*Mellivora capensis*), African wild dog (*Lycaon pictus*), bat-eared fox (*Otocyon megalotis*) [93], Florida panther (*Felis concolor coryi*) [130], and wolverines (*Gulo gulo*) [50]. Serological tests to detect antibodies of other apicomplexan and protozoan parasites have been used to detect *Neospora caninum* antibodies in red foxes, coyotes [97], polar bears [94], and a range of Namibian wild felids and canids [93]. Samples from Namibian wild felids and canids were also used for serological detection of *Besnoitia besnoiti* [93]. ELISA and IFAT for *Leishmania* spp. antibody detection has been implemented in several species of Brazilian wild canids [131].

Few serological tests have been used to detect helminth or arthropod infections in wild terrestrial carnivores. ELISAs developed for the detection of *Trichinella* spp. have been used in different bear species and foxes [94, 132]. Commercially available and laboratory-developed ELISAs for the detection of sarcoptic mange in dogs have been evaluated and successfully used for fox samples [100].

Some serological tests that detect parasite antigens in blood samples have been used to detect parasitic infections in wild canids. ELISAs for the detection of *Dirofilaria immitis* antigen have been used to diagnose coyotes and Island foxes (*Urocyon littoralis*) [92, 116]. An antigen ELISA that detects *A. vasorum* antigen has been evaluated for use in foxes [133]. For these two parasite species, rapid tests based on antigen detection have been developed. The SNAP® 4Dx® Plus test and Angio Detect™ test developed for dogs have been used to detect heart and lungworm infections in coyotes, foxes, badgers (*Meles meles*), and wildcats [19, 134–137]. Compatibility testing and evaluation of these tests with wildlife samples however were done on only a few occasions [19, 134, 135].

#### Serological test evaluation for wild terrestrial carnivores

To the authors’ knowledge so far, no serological tests for the detection of parasitic infections and exposure were developed with wild animal hosts in mind. Serological tests are usually developed to detect parasite antibodies in either companion animals [138–140] or livestock [140–144]. These tests are generally not validated for use in wildlife. Few tests have been evaluated for use in specific wild animal species after their initial development for domestic animals [128, 133, 135]. Serological tests can be translated to wild animal species as they are [145], but occasionally tests must be adapted such as by using species-specific secondary antibodies [146]. The performance of each serological test should be evaluated before its use in serological studies, as validation of the assay, laboratory quality controls, and standards are important for the proper interpretation of serological test results [120]. Interpretation may be challenging as appropriate positive and negative controls may not always be available for a specific animal species or population. To determine appropriate test results sensitivity, specificity, and potential cross-reactions to other parasitic pathogens should be evaluated for each test and each wild animal species [147]. This is best done for individual populations to have population-specific cut-off values, as standard cut-off values are usually not known for wildlife species due to a lack of reference samples from target species and populations [119, 147]. Nevertheless, proper validation of assays for wildlife is often not possible for both laboratory-developed and commercially available tests due to a lack of samples or comparative gold standard tests and data [146].

What further complicates the interpretation of serological results of wild animals are factors regarding seroconversion. Individual immunological responses in wild animals to parasitic infections can vary due to individual fitness and environmental factors [148]. Depending on the animal species and individual there may be variation in seroconversion. Repeated infections may be necessary in some wild animals or species to induce a detectable antibody response. Furthermore, if animals were sampled during incubation time the result may be negative [119]. Therefore, negative results may not necessarily rule out infection nor exposure. On the other hand, positive results may not always imply infection. Many wild animals are coinfected with multiple parasite species, which may lead to cross-reactions in certain serological tests [119]. When testing very young animals the chance of detecting maternal antibodies needs to be considered [149]. Generally, the half-life of antibodies to specific parasites and the duration of their detectability is not known for most wildlife species and may even differ in individual animals or populations [133]. These all represent notable limitations of serological methods for confirmation of parasitic infections in wildlife and may lead to misinterpretation of serological data. Therefore, for each sample acquisition method and each serological test strengths and limitations need to be considered to accurately interpret the results of wild terrestrial carnivores.

## Analysis of tissue

### The importance of the time of death

The quality of tissue sampling obtained from a cadaver is greatly affected by the time an organism dies [150]. It is commonly accepted that the broader the time since death is, the capability to estimate the postmortem interval (PMI) with accuracy reduces significantly [151–153]. It requires significant caution to accurately answer the question of when the exact moment an individual died happened. That is important when dealing with criminal cases (i.e., murder) where the impact of a correct evaluation could suggest or remove suspects and validate or invalidate a suspect’s alibi [151, 153, 154]. Hence, identifying the cause of death may play a serious role when it comes to tissue degradation. Since wild carnivores often compete with humans for resources and prey on domestic herbivores, unnatural death can be often observed [155–157].

Death will lead to a series of irreversible and unavoidable changes that will take place with a constant progression, even though the rate of these modifications can significantly vary due to a wide range of environmental and circumstantial factors [150, 158]. Although the estimation of the time of death has been a topic of countless papers, a final accurate answer does not exist so far [150, 158, 159]. It is clear that the decomposition rate is a multifactorial process related to abiotic factors such as temperature [160–162] or humidity [163–165] and biotic factors such as bacteria [166–168] or insects [169–171]. Understanding the correlation between the location of a carcass found, along with the ecosystem in this place and inside the carrion itself, is valuable and can shed light on different aspects, such as the animal's origin [157, 172].

Nowadays, it is still commonly accepted that, occasionally, the best way to establish the time of death is via forensic entomology [159]. The latter is a forensic science that uses insects and other arthropod evidence to obtain the PMI by the time insects colonized the carcass [173–175] and by evaluation of their expected lifecycle [177] along with the size and instar (age) of larvae [175].

Forensic entomology uses the understanding that diverse organisms will arrive at a carcass in different time intervals [176]. Among the most important species, flies play a crucial role in carcass degradation [176, 177] and are even considered stereotypical arthropods [178]. The first flies arriving at the carcass are blowflies (family Calliphoridae) along with flesh flies (family Sarcophagidae) [176, 177]. Aiming to find a suitable medium to lay their eggs or deposit their larvae rather than feed on, adult female blowflies and flesh flies are attracted to a fresh carcass. The female blowfly will oviposit large clusters of eggs (approximately 200 eggs each time), while the female flesh fly will larviposit on the remains [176, 178]. The eggs will hatch into the first larval stage at an expected interval [176, 178]. As the first instar feeds on the tissue, it will molt into the second, and then into the third larval stage [176, 179]. After that, the instar enters a wandering period in which it quits feeding and migrates far from the carcass to a dry and protected area to pupate [176, 177, 180] until molting and coming out as an adult fly.

Insects do not provide the actual time of death but rather the minimum postmortem interval (PMI_min_) [180–182]. PMI_min_ is the determination of the amount of time that a carcass has been exposed to insect colonization. The maximum postmortem interval (PMI_max_) is mainly in use in human cases, and it refers to the estimated time that the person was last seen alive [173, 181, 182], and therefore, less applicable in veterinary medicine cases.

Sadly, forensic entomology is widely underused in wildlife legal investigations, although it is a well-established discipline commonly used in police investigations of human cases. There is a significant gap in knowledge of the field among the authorities responsible for wildlife [182]. Yet, on many occasions, insect evidence has similar value in human and wildlife cases [182]. The insects associated with a carcass can assist with analyses related to the PMI and may also indicate whether the carrion has been relocated [182]. Moreover, insects and other parasites could provide clues regarding the animal’s origin [175, 182]. Nonetheless, a careful examination of the larvae is required since some species could colonize an organism intra vitam and, in such cases, be considered myiasis.

Myiasis can be classified into three categories. Obligate myiasis is when the insect has a period during its lifecycle for which it depends entirely on a living host (true parasitism) [163]. While many fly types induce true parasitic myiasis in domestic herbivores, the data regarding wild carnivores is poor and involves a narrow range of fly and host species [184–186]. Usually, obligatory parasites damage the surrounding tissue less when compared to facultative myiasis [176, 182]. Facultative myiasis is when insects that normally colonize carrions (mainly blow flies) colonize wounds or traumatized tissue. This is the most prevalent form of myiasis and the most significant in terms of forensic entomology [163, 176, 183]. Contrary to obligatory myiasis species, which are true parasites, the flies involved in facultative myiasis do not depend on the living host nor living tissue [183]. Cutaneous myiasis caused by facultative myiasis has the most damaging effect on the organism since, from an evolutionary perspective, the host being dead or alive will not affect the insect [183]. Accidental myiasis or pseudomyiasis occurs when dipteran eggs or larvae are accidentally eaten or licked from a wound [176, 183]. The insect species involved do not need a host to complete their life cycle [183].

To assume the PMI_min_, forensic entomologists estimate the age of the oldest immature insects [183]. Probably, an animal colonized during life died later [176, 183]. This colonization can occasionally occur much earlier than the actual time of death [183]. This fact raises significant problems since it might wrongfully suggest that the animal was colonized after death, resulting in an overestimation of the PMI [176, 183].

Wild carnivores are more commonly investigated for parasites using parasitological necropsy [3], which involves macroscopic inspection for the detection of visible parasites such as ectoparasites (ticks, fleas, lice, Hippoboscidae) [187, 188] or endoparasites (adult helminths) and microscopic techniques for the detection of specific localized parasites (e.g., *Trichinella* spp. larvae in muscles or *Otodectes cynotis* mites in the ear canal) [189] or even complementary techniques like histological examination, serology, or DNA analyses using specific tissue samples [190, 191]. However, the parasitological investigation correlates with the general state of the carcass, and the diagnosis should be based on all aspects that can influence the results.

### Sample collection and preservation

**Fig. 5:**
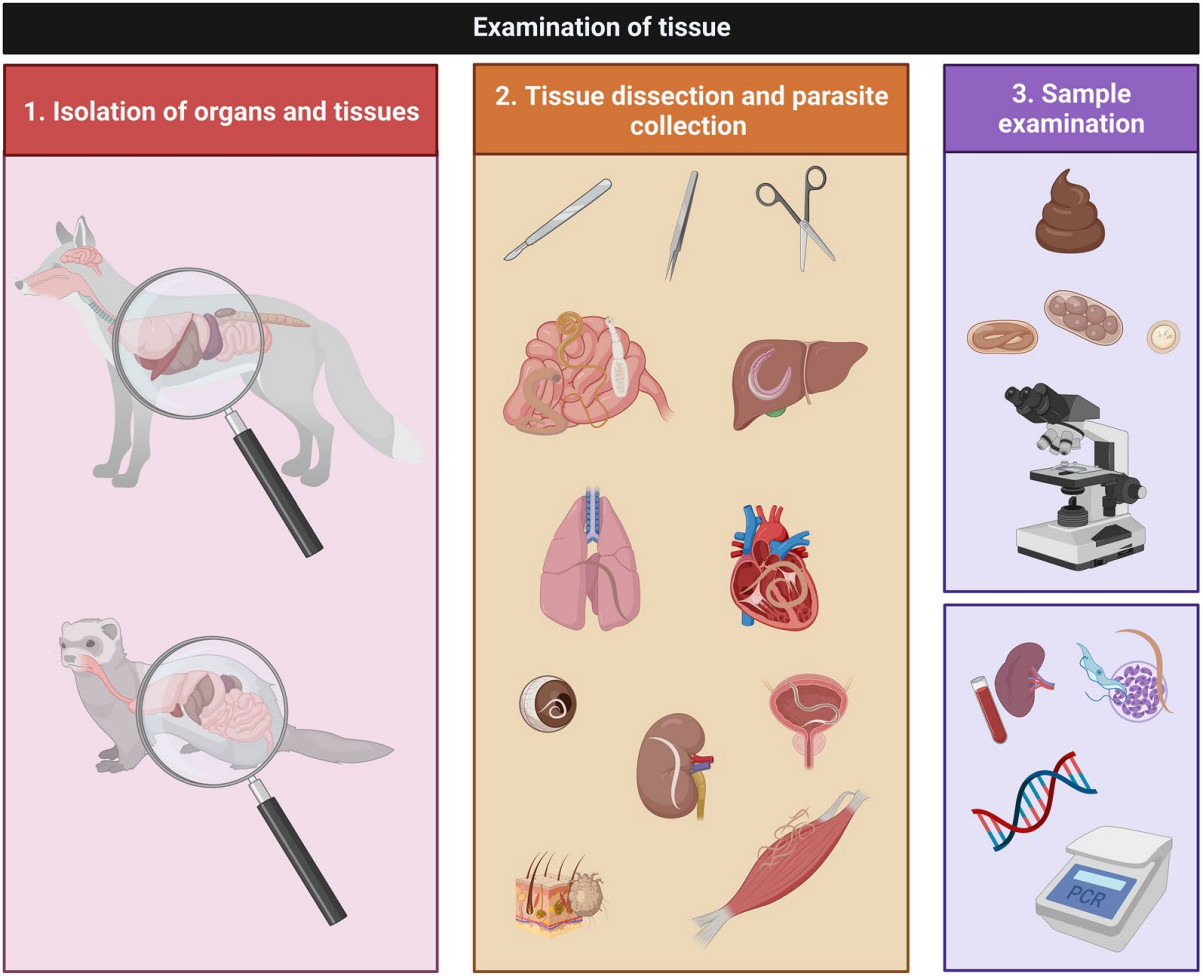
Basic procedures for the analysis of parasites associated to tissue samples from wild terrestrial carnivores. This figure was created using Biorender.com

Tissue samples can be harvested from freshly collected/hunted carcasses or after freezing, which is used for the conservation of the carcass (Fig. 5). Commonly, samples are collected after freezing to avoid biosafety issues and migration of ectoparasites away from the host [52]. Depending on the targeted type of examination, there are several possibilities for collecting and preserving tissue samples depending on the needs, physical space and volume of samples, and possible destination of samples (if shipping is involved).

Commonly, when an entire carcass is available, two to three tissue samples from all organs are collected. Samples can be preserved in labeled plastic bags, cryotubes, or other types of recipients and directly frozen or maintained at room temperature if placed in 10% formalin or absolute ethanol [192, 193]. Freezing has the advantage of keeping the samples suitable for multiple types of techniques (DNA isolation, serology, microscopy) for long periods of time. But, it requires a lot of space in high-volume institutions, and it is more difficult to transport them if needed. Absolute ethanol-fixated tissue fragments can be used for multiple DNA isolations and kept either at room temperature or in the freezer for long periods. This kind of conservation is mainly used for molecular investigations of parasites [194].

Formalin fixation is unsuitable for DNA isolation due to DNA degradation [53]. However, when it comes to the morphological identification of preserved specimens or carcasses in formalin, this method is widely used, especially in museums, for long-term preservation and proved very reliable for endo- and ectoparasites [194]. In addition, the preservation of tissue samples in formalin is required for histology, an additional method of diagnosis. In this case, samples are placed directly in formalin and can be kept for several years. It is worth noting that it is recommended to change the formalin periodically to improve tissue preservation. A second frequently used option is to embed tissue samples in paraffin blocks immediately after formalin fixation. This method comes with two major advantages: the easy long-time dry room temperature conservation method and the possibility of DNA isolation (thus challenging) from the tissue sample [194]. Even though it is possible to obtain genetic information from formalin-fixed tissues containing parasites, there are very few available sequences for comparison for parasites of wildlife [195]. In addition, paraffin blocks are easy to transport and require a minimum of paperwork when shipped internationally.

Other long-term room temperature preservation methods were also described with good results for obtaining genetic material. Caputo et al. [196] compared three different media for the conservation of soft tissue and successfully isolated DNA from all of them. However, after 1 year, samples preserved in NaCl were dried, those kept in garden soil had fungal growth, and untreated samples became liquid due to autolysis [196]. Parasite genetic material was obtained successfully from tissue from human mummies for *Trypanosoma cruzi* [197], *Schistosoma mansoni* in liver tissue [198], and *Leishmania tarantolae* [199]. It is challenging to examine highly putrefied carcasses for parasites. To date, there are no reports of parasites or other pathogens diagnosed by PCR from putrefied carcasses, even though several studies investigated the action of putrefaction on DNA recovery in human legal medicine [200, 201].

### Tissue examination for detection of parasites

No matter the carcass conservation technique and its general state (fresh/frozen/rotten), the examination should always start with the presence/absence of lesions and determination of the type of lesions (antemortem versus postmortem), which requires specialized skills.

**Fig. 6:**
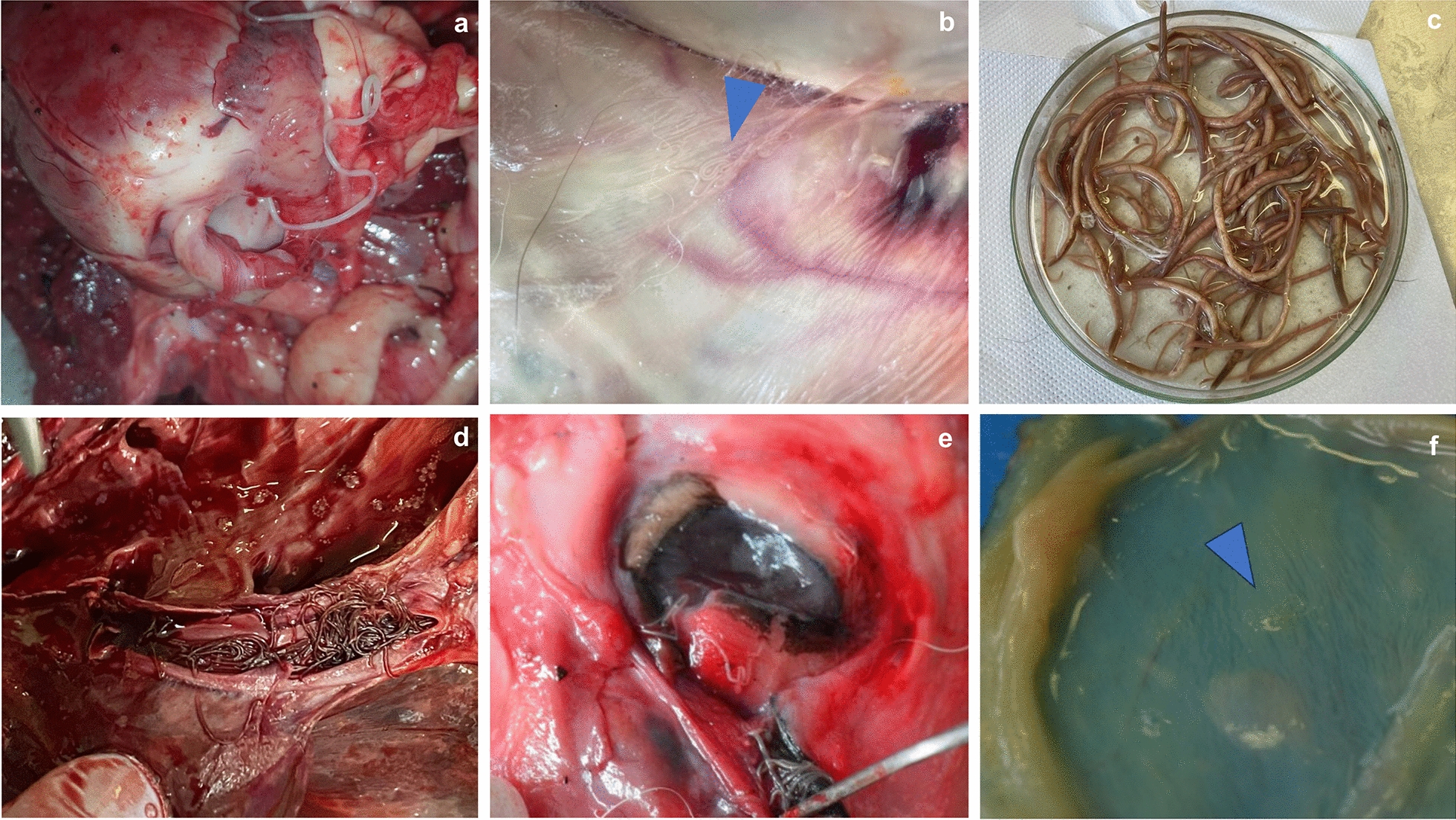
Examples of endoparasites detected during necropsy. **a** *Dirofilaria immitis* in the heart of a golden jackal (*Canis aureus*). **b** Subcutaneous *D. repens* in a golden jackal (*Canis aureus*). **c** Macroscopic appearance of *Baylisascaris transfuga* collected from a brown bear (*Ursus arctos*). **d** *Angiostrongylus vasorum* in the pulmonary arteries of a red fox (*Vulpes vulpes*). **e** *Thelazia callipaeda* in the eyes of a golden jackal. **f** *Pearsonema plica* in the urinary bladder of a stone marten (*Martes foina*)

The examination depends on the type of parasite to be identified and the location in the host (Fig. 5). Macroscopic parasites can be easily identified in organs using simple dissection techniques. Usually, necropsy starts with skinning during which subcutaneous parasites such as *Dirofilaria repens* [202] (Fig. 6b), *Filaria martis* [203], or even ticks [204] can be detected in wild terrestrial carnivores. After detection, helminths can be removed with fine tweezers and preserved in formalin, ethanol, or both for further identification.

For the detection of adult cardiorespiratory parasites (Fig. 6a, d), the entire cardiorespiratory tract should be removed and isolated, then each segment is sectioned longitudinally starting from the trachea to the alveolI followed by the pulmonary artery and the heart chambers, then a macroscopic examination is conducted. For better visualization of small specimens, it is highly recommended to examine the tissue under a stereomicroscope. Several complementary methods can be used to prevent false negative results. In addition to sectioning, the lung parenchyma can be immersed in water and squeezed several times, and the obtained sediment examined under a stereomicroscope [205]. A similar method for parasite collection is the lung flushing or lung perfusion technique with PBS or saline solution, which is also used for the collection of nematodes with vascular localization (e.g., *A. vasorum*) [206, 207]. Other authors also recommend the artificial digestion of lung tissue, which proved to be of great use for *A. vasorum* extraction [208] and even liberation of very long and coiled *Filaroides osleri* adults from nodules for a better morphological identification (personal observation of the authors). For the detection of small and profound parasites (e.g., *Aelurostrongylus abstrusus* in cats) small tissue sections can be collected and boiled in lactic acid followed by tissue compression between two glass slides and examination under a microscope [209]. This last method has the advantage of the possibility of adding Canada balsam and conserving the specimens for years. Besides adult parasites, larval stages can be detected in the respiratory tract using the Baermann method from lung tissue [210]. Although this method should be used in fresh carcasses, some larvae are very resistant and still motile even after deep freezing for many months (e.g., *C. vulpis*) and others can be collected dead by gravitational force [211]. Histopathological examination proved to be a key method for the postmortem detection of lungworms in wild carnivores, as it was shown for *Troglostrongylus brevior* in the Eurasian lynx and *A. vasorum* in wolves [212, 213]. Eggs and larvae can be detected using tracheal or bronchial scrapings as well as histopathological examination [206]. Microfilariae can be detected in the blood or in sanguinolent body fluids collected from carcasses using the modified Knott’s test [214] with distilled water [215].

Wild carnivores can be commonly infected with parasites localized in the nasal cavities (e.g., *Linguatula serrata*, *Eucoleus boehmi*, *Troglotrema* spp.) or sinuses, which are more difficult to observe. A commonly applied method for their detection is the sagittal cutting of the heads, removal of the content with a scalpel, and direct examination [21, 216]. Barton et al. [216] described a technique for the collection of *L. serrata* with the preservation of the intact skull. This technique, however, may not be suitable for thin nematodes such as *E. boehmi* or *E. aerophilus* because of the possibility of aspiration during death or the localization in the sinuses.

In muscle tissue, microscopic parasites such as *Trichinella* spp. larvae or *A. alata* mesocercaires can be identified using muscular sections and examination between two glass plates, by artificial digestion, or histology [189, 217, 218]. Artificial digestion is more sensitive, however, when working with frozen carcasses, larvae may lose their motility, but they are still released from the tissue [219].

For the detection of digestive parasites, the entire digestive tract should be carefully examined, starting from the buccal cavity to the rectum, using visual inspection and complementary methods. Postmortem, the digestive tract should be isolated and removed from the carcass and further divided into segments. Each segment should be medially opened from the tongue to the anus, and macroscopic parasites like ascarids, strongyles, acanthocephala, and some cestodes can be visualized and collected [220] (Fig. 6c). However, small nematodes (e.g., *Strongyloides* spp. or *Trichinella* spp.) and certain cestodes (*Echinococcus* spp.) are not always visible especially when there is hemorrhage or intestinal content. For the detection of small parasitic forms, mucosal scrapings followed by sedimentation and examination for parasites under the stereomicroscope or successive washings of the digestive content in sieves and examination can be done [221]. Some authors prefer a different method, using the artificial digestion of each isolated digestive segment. The last, however, implies the use of chemical substances, which can be time consuming and expensive, but it is suitable for releasing encysted parasitic forms [222]. Intracellular forms of protozoans like *Cystoisospora* spp. can be detected using the mucosal wet smear method [223] or histology [224], the last being unsuitable in frozen carcasses. In addition to the direct examination, coproscopical methods can be done using feces collected from the rectum or even gastric content for the detection and identification of preimaginal parasitic forms [225, 226].

Less commonly investigated helminths such as the giant kidney worm *Dioctophyme renale* [227] in the renal capsule or *Pearsonema plica* and *Pearsonema feliscati* in the urinary bladder [228] can be detected by dissection of the parasitized organs (Fig. 6f). In the case of *Pearsonema* spp., worms can be missed as they are small and thin, and diagnosis should be complemented by mucosal scrapings or urine sediment analysis for the identification of their specific eggs [228].

Although not very common, liver trematodes were detected in wild carnivores on several occasions using a standard dissection method [187, 229, 230]. For more accurate detection, the gall bladder can be opened under a stereomicroscope and scrutinized for parasites or artificial digestion of the entire organ can be done [230].

Ocular parasites are easy to observe depending on the location. *Thelazia callipaeda*, the oriental eye worm, was reported in free wild carnivores [13] and more recently in zoo carnivores [231]. This nematode is usually diagnosed by the visual inspection of the eyes, especially under the nictating membrane (Fig. 6e). A less frequently reported nematode in wild carnivores is *Onchocerca lupi* [232]. This nematode has a more profound ocular localization in various regions of the eye (cornea, retrobulbar space, conjunctiva) where it forms nodules. Considering this, the detection of these nodules involves the complete removal of the ocular globe and careful examination [233]. For larvae detection, skin tissue samples can be collected from the interocular frontal area of the head and immersed in a warm saline solution. The sediment is then examined under light microscopy [234]. Other ocular parasites with ectopic migrations were reported in wild carnivores and reviewed in Otranto and Deplazes [10]. Using skin biopsy, microfilariae of other skin-dwelling nematodes (i.e., *Cercopithifilaria*) can be found [235]. Hence, precise morphologic and morphometric examination is crucial. After the complete removal of all internal organs, the empty carcass should also be inspected for eventual encapsulated parasites or cysts (e.g., metacestodes) [236].

**Fig. 7:**
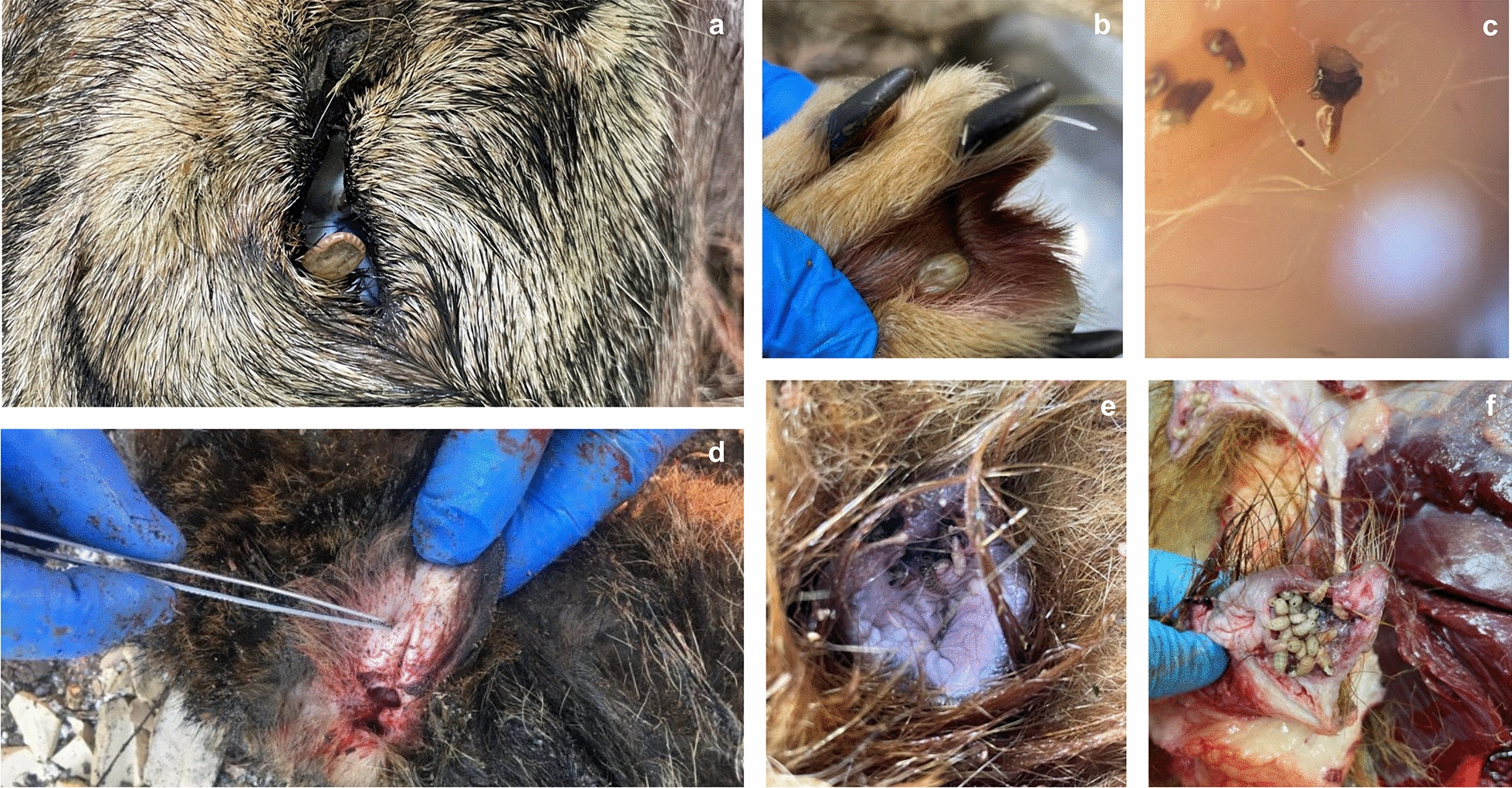
Examples of arthropods detected during necropsy. **a** Tick fixed in the eye region of a gray wolf (*Canis lupus*). **b** Tick fixed in the interdigital space of a gray wolf. **c** Subcutaneous tick in an African wolf (*Canis lupaster*). **d** Tick fixed on the internal part of the ear pavilion in a golden jackal (*Canis aureus*). **e**, **f** Myiasis in a Syrian brown bear (*Ursus arctos syriacus*)

Wild terrestrial carnivores are subject to infestation by different ectoparasites, such as fleas, ticks, lice, mites, and others [204, 237]. Postmortem collection of ectoparasites from wild carnivores is influenced by the time between death and collection of the carcass, body temperature, and activity of non-parasitic arthropods [238]. The first step in the detection of ectoparasites involves a macroscopic inspection of the entire body. When active ectoparasites are observed (e.g., fleas), chemical ectocides substances or even normal tap water can be used to inactivate specimens to avoid losing them. Depends on the type of carnivore species ectoparasites can either be combed out (when the fur type and state allow this) or manually collected with fine tweezers [236, 237] (Fig. 7a, b, d–f). Many types of utensils can be used for ectoparasite collection, including brushes, combs, forceps, tweezers, adhesive tape, or even the vacuum method [239]. The vacuum method has the advantage of covering the whole surface of the animal and collecting the majority of ectoparasites. However, when used on a road-killed carcass, the presence of liquid blood and other body fluids from the fur can make the examination more difficult as it colors and wets the textile sheet (personal observation of the authors). For mites with a specific profound localization, such as *Demodex* spp., profound scrapings are needed from various body regions [240–242]. Thick skin crusts can be digested in 10% KOH to release any parasitic forms and further examined under the microscope [243]. Mites with a more internal localization like the ear meatus (e.g., *Otodectes* and *Melesodectes*) [244, 245] or the nasal cavities (*Pneumonyssoides caninum*) [246] can be detected by examining either a cotton swab or slide under the stereomicroscope or by performing the flushing method [247].

### Detection of parasites using molecular biology techniques

Tissue samples are frequently used for the detection of microscopic parasites or other pathogens. Spleen tissue samples represent a good alternative to blood samples for the detection of blood-borne pathogens in wild carnivores. The spleen along with blood are the most commonly selected samples that are suitable for molecular detection of vector-borne pathogens (VBPs) in wild carnivores [109, 248, 249]. The spleen has a high density of nuclei and was used for the identification of Anaplasmataceae, *Rickettsia* sp., *Bartonella* sp., *Coxiella brunetii* [89], and parasites like filaroids [202], or *Hepatozoon*, *Babesia*, and *Cytauxzoon* [250, 251], and *Leishmania infantum* [252]. Other types of tissue can be used for DNA isolation of parasites or other pathogens like the brain for identification of *N. caninum*, *T. gondii*, and *Encephalitozoon cuniculi* [253]. Lung parenchyma is commonly used for the identification of respiratory parasites including migrating nematode larvae using molecular techniques [213]. Muscle sections from various musculature are frequently used for the identification of *Trichinella* [254], *T. cruzi* [255], and *T. gondii* [256]. Commonly, for the identification of *T. gondii*, brain tissue is preferred [257]. Isolation of DNA for detection of parasites or other pathogens is possible from a wide range of tissues, including the skin, bone marrow, nasal and ocular swabs, lymph nodes, and urinary bladder [191, 258]. Tissue samples used for molecular parasite detection methods can either be stored in 70% ethanol or kept frozen [103].

### Serology from tissue samples

Samples collected from tissue can be used for serological testing. Freezing and thawing of organs and tissues, such as plucks, or muscle tissue has been implemented to collect bloody transudate or tissue fluid containing antibodies which can be used for serological testing [21, 259, 260]. Another technique described is placing lung tissue in PBS for the collection of lung transudate-containing antibodies [261, 262]. Lung transudate samples diluted in PBS, however, may result in false negative results due to the dilution of low antibody levels [261]. In case no blood or serosanguineous fluid can be collected from a carcass, bloody tissue fluids represent an alternative to collecting samples for serological testing. Even if carcasses, organs, or tissues were frozen or stored for extended periods sample collection from tissues should still be possible and result in adequate samples for serological testing [260, 263].

## Conclusions

Wild terrestrial carnivores can offer crucial information about parasitic diseases in specific geographical areas. Given the increasing changes in habitat, forest fragmentation, and urbanization, screening wildlife populations for parasitic infections and other zoonotic pathogens is of great relevance to domestic animals and human protection. Nevertheless, studying parasites associated with wildlife offers a different scenario to that from parasites collected from domestic animals or humans. Sampling methods, analysis tools, and several limitations are encountered during field work which are explained in detail herein. The authors advocate for the importance of using adequate diagnosis techniques for the identification of parasitic infections from feces, blood, and tissue samples. This paper gathered available information for parasite investigation in wild terrestrial carnivores.

## Data Availability

All data generated or analyzed are included in this publication.

## Abbreviations

BCM: Buffy coat method
bp: Base pairs
CATT: Card agglutination test for Trypanosoma
*cox*1: Cytochrome oxidase subunit 1
*cyt*B: Cytochrome B
DNA: Deoxyribonucleic acid
EDTA: Ethylenediaminetetraacetic acid
ELISA: Enzyme-linked immunosorbent assay
HCT: Hematocrit concentrations technique
IFAT: Immunofluorescent antibody tests
mAECT: Mini anion-exchange centrifugation technique
mtDNA: Mitochondrial DNA
PBS: Phosphate-buffered saline
PCR: Polymerase chain reaction
PMI: Postmortem interval
PMImax: The maximum postmortem interval
PMImin: The minimal postmortem interval
SAF: Sodium acetate-acetic acid-formalin
SNPs: Single nucleotide polymorphisms
VBPs: Vector-borne pathogens
